# Endoscopic Lumbar Disc Surgery Experience with the TESSYS Technique in 253 Case Series

**DOI:** 10.3390/jcm13071911

**Published:** 2024-03-26

**Authors:** Aldo Spallone, Roman Vladimirovich Khalepa, Evgeniya Amelina, Amrakh Magerramov Asif ogly

**Affiliations:** 1 NCL-Neuromed, Institute of Neurological Sciences, 00178 Rome, Italy; 2 Institute of Bio-Organic Chemistry, RAS Russian Academy of Sciences, 117997 Moscow, Russia; 3 Faculty of Medicine, MSU Lomonosov University, 119991 Moscow, Russia; 4 FSBI “Federal Neurosurgical Center”, 630087 Novosibirsk, Russia; r_halepa@neuronsk.ru (R.V.K.); amrah_m_a@mail.ru (A.M.A.o.); 5 Center for Technology, Transfer and Commercialization, Novosibirsk State University, 630090 Novosibirsk, Russia; e.amelina@nsu.ru

**Keywords:** case series, TESSYS, endoscopic percutaneous, lumbar discectomy

## Abstract

**Background**: Herniated lumbar disc (HLD) is a widespread medical problem which can require surgery. Minimally invasive surgical management can represent an extremely valuable option for patients suffering from HLDs. Transforaminal endoscopic lumbar discectomy is an alternative to classical microdiscectomy which was proposed more than two decades ago and has evolved technologically with time. **Methods**: The transforaminal endoscopic spine system (TESSYS) technique has been introduced in recent years and offers the advantage of performing a controlled foraminal augmentation with full nerve root protection. We started using this technique in 2016 and prospectively evaluated the results of endoscopic TESSYS-based operations performed in a three-year period until the end of 2019. Selection criteria were very strict, and we included only patients with unilateral radicular pain with no instability who failed conservative therapy. Out of the 253 patients operated on in that time span, 183 were available for follow-up evaluation. **Results**: After surgery, there was a clinically significant improvement of all symptoms which basically lasted in the long-term follow-up. Complications were limited and generally minor. Redo surgery with microdiscectomy was required only in four cases. Obesity did not play a clear negative role in patients’ outcome. **Conclusions**: Endoscopic transforaminal discectomy with the TESSYS technique represents a valuable management option for patients harbouring unilateral herniated lumbar disc located laterally.

## 1. Introduction

Sciatica is a socially relevant problem affecting up to 1.5% of the population in developed countries [1,2,3]. The vast majority of patients harbouring a herniated lumbar disc (HLD) are successfully treated through the various technological possibilities ranging from wearing a corset to appropriate physiotherapy, acupuncture, robotics, etc. However, a significant number of patients fails conservative treatment and require surgery [4].

Surgical treatment of HLD was introduced in the mid-1930s and has been refined with time [5,6,7,8,9]. Lumbar microdiscectomy is considered the gold standard for surgery requiring HLD, and its results are good in over 90% of the patients. However, the search for reduced invasiveness has supported using even less traumatic instrumental technology such as endoscopic surgery.

Since Kambin introduced the idea of using an arthroscope for removing lumbar herniated discs [10], several techniques of endoscopic lumbar disc surgery have been suggested such as the Young Endoscopic System (YESS), the Transforaminal Endoscopic Spine System (TESSYS), and the Percutaneous Endoscopic Inter Laminar Discectomy (PEILD) [11,12]. The latter is actually an equivalent of the classical microdiscectomy with reduced invasiveness due to the use of endoscopic instruments.

Several papers have been recently published in regard to the use, the techniques, and the results of endoscopic lumbar disc surgery. A comprehensive meta-analysis has appeared recently in a highly ranked international journal devoted to spinal surgery [13,14,15,16,17,18,19]. These analyses reported the results of spinal endoscopic vs. open surgery in an extensive number of well documented cases, and concluded that endoscopy and microdiscectomy would bring the same results in operated HLDs. However, no comparison has been made in regard to the different surgical techniques used for endoscopic lumbar disc removal.

Among the transforaminal endoscopic techniques, TESSYS seems to offer concrete advantages due to the fact that foraminoplasty definitely increases the angle of view of intraspinal pathology and allows better control of migrated disc fragments [20,21].

TESSYS technology is mainly based on the principles of using several atraumatic reamers which increase the working angle and the working space inside the lumbar canal while, at the same time, the working cannula protects the affected nerve root. As stated above, this concretely increases the indications for its use in lumbar disc disease [22,23,24].

We started using the TESSYS technique in Rome and in Novosibirsk in 2016. Treatment philosophy and surgical indications have been remarkably similar in our centers; thus, we decided to perform a joint prospective study on this surgical resource.

## 2. Materials and Methods

### 2.1. Study Design

The present study was conceived during a period in which the first author (AS) was the visiting Professor in Novosibirsk (2016). The leading surgeons in Rome (AS) and Novosibirsk (RK) had undergone full training with the TESSYS technique after having developed extensive experience with spinal microsurgery. Both required approximately 4 to 6 months to feel fully comfortable with the new technique, and to be able to perform endoscopic surgery with sufficient confidence. Criteria for the selection and objective clinical evaluation of study patients were agreed and implemented at the same time in both institutions.

Patients of this case series were operated on in both Rome and Novosibirsk Institutions during a three-year period starting in 2017, using the TESSYS technique. Indication for surgery was placed after an unsuccessful course of conservative therapy for at least 6 weeks.

The patients were prospectively included in the present investigation. The study was approved by both ethical committees (protocol n. 1811.2015 in Novosibirsk, date 18 November 2015, Federal Center of Neurosurgery, Novosibirsk, Russia; protocol RM2-NCL n 2017-003512-20 Rif 156/17 in Rome, date 17 January 2017 USL RM2, Rome, Italy). The study was conducted in accordance with the Declaration of Helsinki. Patients were fully informed about the fact that the TESSYS technique was brand new, as well as of its pros and contras, and agreed to participate.

Criteria for inclusion were the presence of symptoms of unilateral root compression which matched well with preoperative diagnostic neuroimaging, CT scanning (143 cases), and MRI (all 253 cases). CT scanners were 64-slice machines and MRI machines were 1.5 Tesla machines in both institutions. Novosibirsk mounted Siemens scanners and Rome GE scanners were used. In cases in which the diagnosis was doubtful, a diagnostic selective nerve root block was performed in order to identify the affected nerve root requiring decompression.

The exclusion criteria were ossified disc herniation, lumbar spinal stenosis, migrated disc herniation, central disc herniation, segmental instability, prior surgery at the same segment, cauda equina syndrome, spinal tumors, or infections.

A total of 70 patients (27.6%) were lost during the 5-year follow-up period. Complete data could be collected from 183 (139 Russian and 44 Italian) patients.

### 2.2. Patient Data

Clinical features before, the day after the surgery, and during the follow-up period were evaluated using the Visual Analogue Score (VAS) and Oswestry Disability Index (ODI). Body Mass Index (BMI) was also taken into account.

The mean operation time was 67.6/60 (45–90) min. Hereafter, the data format is mean/median (1st–3rd quartile). The mean hospital stay was 2.7/3 (2–3) days. Patient demographics are shown in Table 1.

### 2.3. Surgical Data

**Figure 1:**
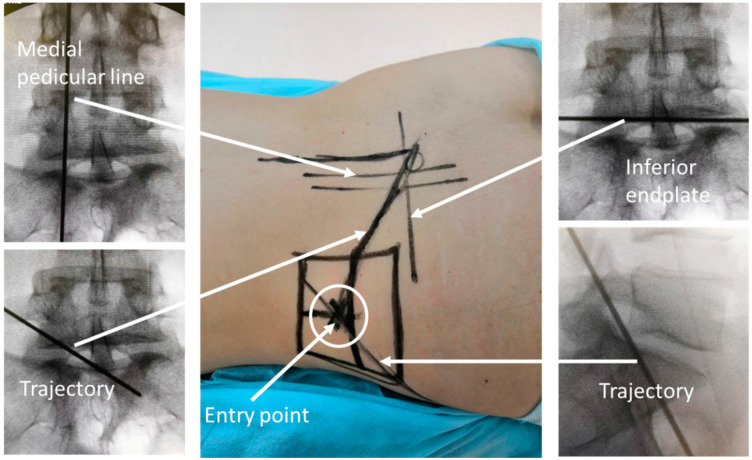
Preoperative planning.

Surgical procedures were performed in the prone position under general anaesthesia using a foraminoscope of the TESSYS system with an optic angle of 25 degrees. The entry point was 8–14 cm lateral to midline and determined by preoperative planning and intraoperative C-arm fluoroscopy (Figure 1). 

**Figure 2:**
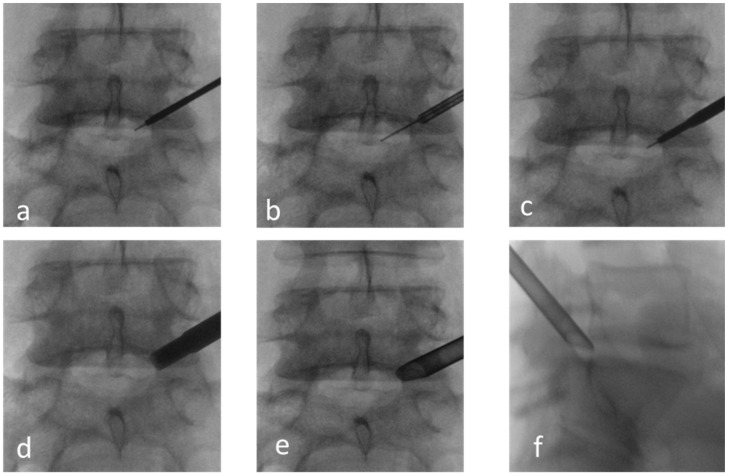
Approach to the spinal canal through foramina. (**a**–**d**) Insertion of a guide wire, dilators, and reamers into the foramina. (**e**,**f**) X-ray checking of working channel position.

An 18 G needle was inserted into a spinal canal so that the tip of the needle crossed a medial pedicle line in a posterolateral view but did not cross posterior border of the disc in the lateral view. Then, step by step, a guide wire and tubular dilators were inserted. Neural foramina were widened with tubular reamers. Then, a working cannula was inserted over the dilator through the foramina into the epidural space (Figure 2).

**Figure 3:**
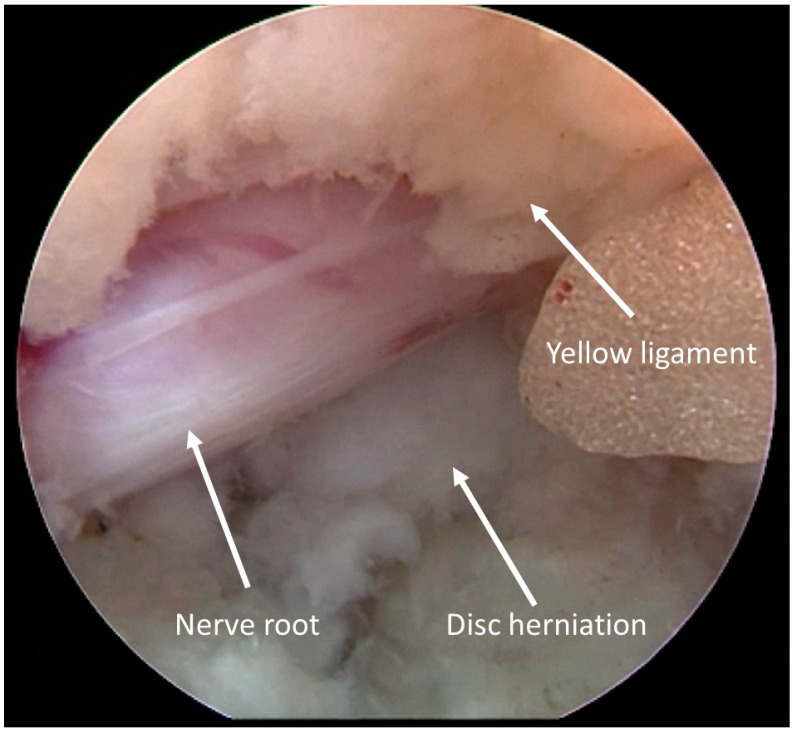
Intraoperative view. Disc herniation under the nerve root.

The proper position was checked by fluoroscopy; then, an endoscope system with irrigation and working channels was introduced and used for the next steps (Figure 3).

**Figure 4:**
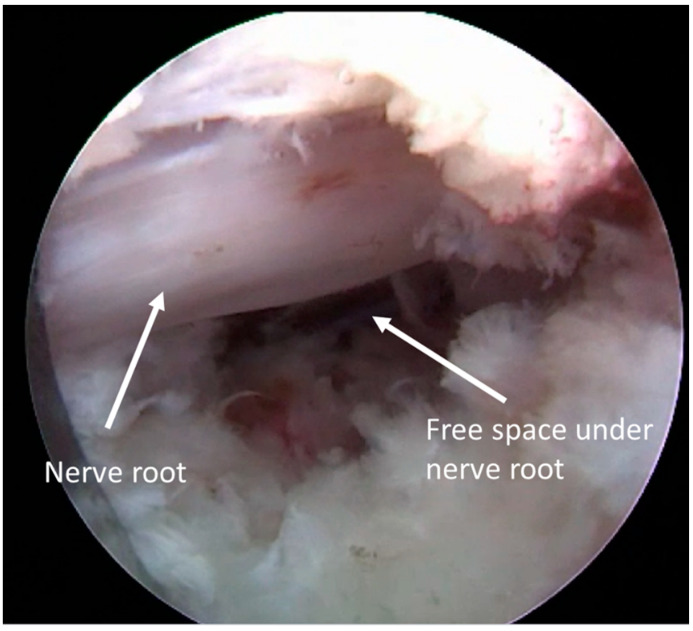
Decompression criteria.

Diamond burr was used for foramina dilation and superior facet resection if necessary. The herniated disc was dissected by a bipolar probe and removed with punches out of the epidural space. When the decompression criteria were reached (free space below nerve root and its pulsation and floating in the epidural space), the procedure was considered completed (Figure 4). Hemostasis was checked, and the endoscope was then removed.

The distribution of operation levels is shown in Table 2.

### 2.4. Statistical Analysis

We observed a nonnormal distribution of most values (Kolmogorov–Smirnov test). In the present paper, the numerical data format is as follows: mean/median (quartile 25–75%). Comparison of pre- and postoperative parameters was performed using a two-sided Wilcoxon signed rank test with continuity correction. R software (version 4.3.3) was used for statistical data processing (R Core Team R: A language and environment for statistical computing. R Foundation for Statistical Computing: Vienna, Austria (2020). http://www.r-project.org/index.html, accessed on 23 March 2020).

## 3. Results

### 3.1. Clinical Outcomes

The mean follow-up time was 36.1/36 (23–50.2) months. Data are shown in Table 3. The mean VAS of leg pain before surgery was 6.4/7 (5–8) points. Following surgery, the mean VAS of leg pain improved to 0.8/0 (0–1) the next day after surgery and 0.6/0 [0] nowadays at the last follow-up control (*p* < 10^−15^; Table 2). The mean VAS of back pain before surgery was 4/4 [2–6) points. VAS of back pain improved to 1/0 (0–2) the next day after surgery but deteriorated to 1.2/1 (0–2) (*p* < 10^−14^; Table 3) on follow-up. The mean ODI before surgery was 60.8/60 (48–72)%. The mean ODI at follow-up is 6.2/2 (0–10) (*p* < 10^−15^; Table 3).

After surgery, there was a clinically significant improvement MCID (Minimal Clinically Important Difference) in pain in the leg, spine (VAS), and physical activity (ODI). The data are shown in Table 4.

Twenty-nine (15.8%) of our patients were obese, but there was no association of complication rate, hospital stay, or operation time with increased BMI. Only one of these patients required microsurgical reoperation for persisting symptoms following endoscopic discectomy.

### 3.2. Complications

Complications are grouped according to the Clavien–Dindo classification. Despite a significant number of all complications (12%), which included any deviations from the normal process of surgical intervention and the postoperative period, the number of complications that required medical correction (type II) and repeated surgical intervention (type IIIB) is small: 2.7% type II and 6% type IIIB. The proportion of complications that did not affect the quality of life, had no clinical manifestations, and did not require any correction was 3.2%. Cases of recurrent disc herniation and unresolved nerve root compression required revision microsurgical decompression. The highest number of complications was observed in the first 12 months of mastering the technique of transforaminal endoscopic procedures (7.6%). Unintended durotomy and nerve root injury without neurological deficit did not require any treatment. Another complication result, namely root compression and early recurrence of disc herniation, occurred in four cases and required repeated surgery. After 1 year, the number of complications decreased to 4.3%. Data of the complications are shown in Table 5.

Here, we report in brief detail two of the present cases.

### 3.3. Clinical Case 1

**Figure 5:**
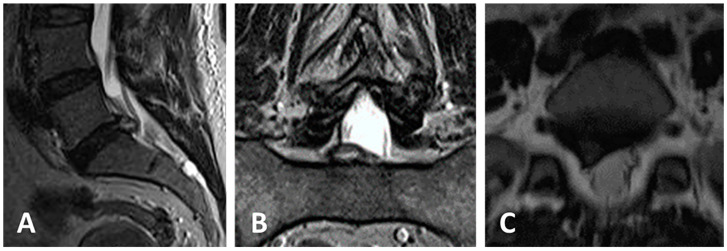
(**A**–**C**) Preoperative MRI shows an extruded disc herniation at the right L5-S1 level. VAS was 6, ODI was 56.

**Figure 6:**
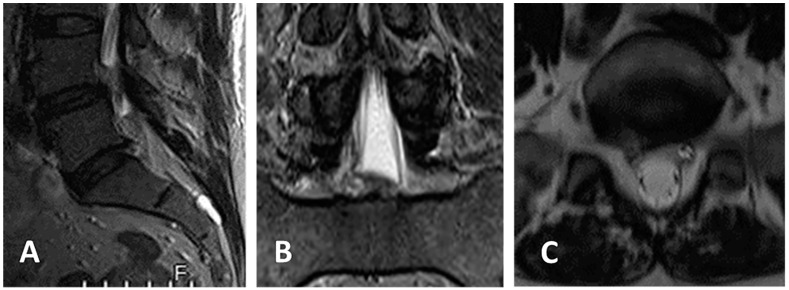
(**A**–**C**) Postoperative MRI on the same day of operation shows complete epidural decompression after removal of the herniated disc with the TESSYS technique. VAS 1, ODI 6.

Case of a 48-year-old female patient presenting with right sciatica on the S1 dermatome (Figure 5 and Figure 6).

### 3.4. Clinical Case 2

**Figure 7:**
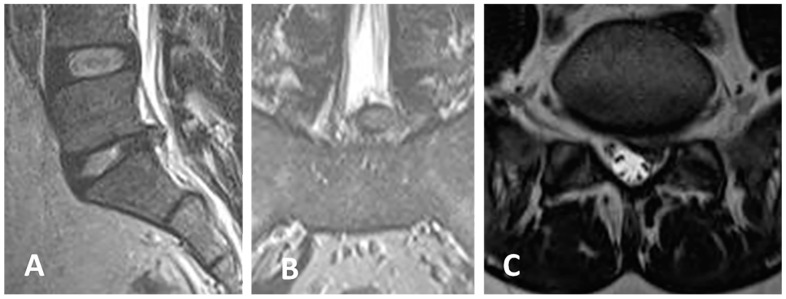
(**A**–**C**) Preoperative MRI shows an extruded disc herniation at the left L5-S1 level. VAS was 7, ODI was 60.

**Figure 8:**
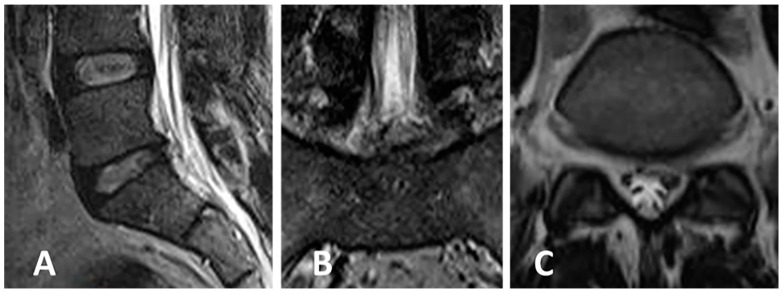
(**A**–**C**) Postoperative MRI (of suboptimal quality since the patient was restless during examination) shows adequate epidural decompression after removal of the herniated disc with the TESSYS technique. VAS leg 0, ODI 4.

Case of a 37-year-old female patient presenting with left S1 sciatica and moderate left steppage. VAS was 7, ODI 60 (Figure 7 and Figure 8).

## 4. Discussion

We analyzed here our experience with 183 patients harboring HLD operated in a three-year period in both Rome and Novosibirsk centers by a transforaminal endoscopic approach using the TESSYS technique.

This technique has been recently reported to be associated with improved results in the treatment of lumbar discogenic disease [12]. By reducing the surgical trauma and its consequences [11], this technique seems to be particularly attractive and has recently received great attention by the community of spinal surgeons [11,12,25]. It requires a steady learning curve which could potentially represent a problem for experienced spinal surgeons well accustomed to traditional open-surgery techniques [11]. However, our personal experience demonstrated that this is quite possible, and adaption to new surgical concepts, new surgical anatomy, and new instrumentation would not be a problem if the experienced surgeon would be patient enough to complete their “re-training” period as indicated.

The TESSYS technique is based on the concept that enlarging the intervertebral foramen if properly planned gives the necessary angle of vision for adequately removing all pathological disc material located inside the spinal canal, while at the same time decompressing the affected nerve roots via a foraminal enlargement [26].

Our experience substantially suggests the same concepts. Endoscopically operated patients show a good early as well as midterm good outcome if they harbor laterally located herniated discs, either with or without having undergoing previous “classical” lumbar operation.

As expected, results are definitely better after a six-to-twelve-month period of clinical experience, as a logical learning curve of any technically innovative surgical procedure. Surprisingly, overweight does not seem to play a negative role as generally observed in studies on lumbar disc diseases patients; actually, out the 29 obese patients operated on with this technique, only 1 complained of persistently significant symptoms following endoscopic discectomy. The present experience shows a low incidence of recurrent relapsed disc following the endoscopy procedure. This incident is low, if compared to the data from recently published meta-analyses [13,14,15,16,17,18,19]. In our opinion, this is not surprising since we choose performing endoscopic removal only in laterally located herniated discs, and we strongly recommend this policy in the patients’ selection for endoscopic lumbar surgery.

The present study substantially shows two facts: a learning curve is unavoidable and clinical results are better in patients operated on six months to one year after the introduction of the TESSYS technique in the clinical practice; the more the disc pathology is located laterally, the better the clinical result. This fact is particularly relevant in patients with residual disc pathology following either a previous “traditional” operation or after instrumentation surgery. In such cases, it can avoid revision of the instrumentation construct, whilst in the former scenario it can prevent struggling with surgical scars [27].

Centrally located disc fragments are difficult to be adequately managed with this technique, since its proper visualization is jeopardized by the angle of view which this technique creates [28]. They can be removed blindly, but it is obvious that a “blind” removal does not guarantee against the risk of leaving some disc material behind. For these reasons, contrary to the opinion of others [29], we do not consider the TESSYS technique for centrally located HLDs and strongly advise open microsurgery for these patients. Neither we would recommend combining YESS and the TESSYS technique for dealing with these problems. We strongly believe that “traditional” lumbar microdiscectomy represents the gold standard and that the TESSYS technique is to be used in those specific situations in which it would bring evident advantages over the classical techniques. Lateral disc fragments affecting the nerve roots at the foramen, with or without concomitant foraminal stenosis, are the main examples of what we believe would be those situations in which the TESSYS technique is definitely more appropriate.

Our clinical results clearly support our philosophy. This means that classical lumbar microdiscectomy is to be considered the gold standard, whilst the TESSYS technique is clearly indicated in those patients in whom foraminal pathology represents the main component of nerve roots compromise. We would like to outline that we did not measure the disc height, pre- and postoperatively, for comparison. We admit that this could represent a shortcoming of the study planning. However, as clearly stated above, we considered for surgery only patients harbouring either laterally or medio-laterally prolapsed discs, and removal of the herniated disc fragment was basically limited to the offending portion of the disc with no attempt to perform an aggressive removal of the non-pathological portion of the diseased disc. Therefore, we did not consider this parameter crucial to the purpose of the present study.

In the present study, we purposely did not consider redo surgery patients as well as those who either previously had or would require lumbar stabilization. However, based on our experience, we strongly believe that patients with residual lateral disc fragments following lumbar microdiscectomy as well as following instrumentation surgery can be excellent candidates for this type of surgical strategy. Moreover, if the clinical situation would require it, stabilization and endoscopic discectomy can be performed in the same session with reduced invasiveness.

As stated above, the learning curve of this technique does not represent a problem even for aged, experienced surgeons. Certainly, the new generations of neurosurgeons will be more exposed and interested to be familiar with this and others modern techniques, and it is very likely that the whole panorama of surgical treatment of spinal diseases will undergo significant changes with the progressive introduction of less invasive procedures [25,30,31,32]. However, in the present scenario, classical procedures still play a major role and maintain their indications. The TESSYS technique, also demonstrated by our experience, can be an extremely valuable adjunct in certain specific cases of HLD in which the foraminal component is prevailing.

## Figures and Tables

**Table 1:** Patient demographics.

	Total	Russian	Italian
Number of patients	183	139	44
Age	39.4/38 (31–46)		
Sex	female—99 (54%), male—84 (46%)	female—75 (53.9%), male—64 (46.1%)	female—24 (54.5%), male—20 (45.5%)
Body mass index (BMI)	28.1/27.4 (23.3–30.9)		
Overweight	29 (15.8%)	18 (12.9%)	11 (25%)

**Table 2:** Levels of surgery.

Level	Number	%
Th12-L1	1	0.5%
L2–3	2	1.1%
L3–4	14	7.7%
L4–5	82	44.8%
L5-S1	84	45.9%

**Table 3:** Patients’ clinical characteristics before and after surgery.

Follow-Up 36.1/36 [23; 50.2] Months					
	Before Surgery (183)	The Day after Surgery (183)	Follow-Up (183) Months	*p* (Before Surgery—The Day after Surgery)	*p* (Before Surgery—Follow-Up)
VAS spine	4/4 (2–6)	1/0 (0–2)	1.2/1 (0–2)	<10^−15^	<10^−14^
VAS leg	6.4/7 (5–8)	0.8/0 (0–1)	0.6/0 (0–0)	<10^−15^	<10^−15^
ODI	60.8/60 (48–72)	-	6.2/2 (0–10)	-	<10^−15^
McNab (excellent, good, fair, poor); number—percent	- -	(77, 95, 7, 2)– (43, 52, 4, 1)%	(53, 67, 18, 1)– (38, 48, 13, 1)%	- -	- -

**Table 4:** Clinical improvement according to MCID.

	Before Surgery—The Day after Surgery	Before Surgery—Follow-Up
VAS leg/MCID	−3.6/−3.8 (−4.4–−3.1)	−3.7/−3.8 (−5–−3.1)
Δ VAS leg < −1.6	93% (170 of 183)	93% (170 of 183)
VAS spine/MCID	−2.5/−2.5 (−4.2–−0.8)	−2.2/−2.5 (−4.2–0)
Δ VAS spine < −1.2	67% (122 of 183)	66% (121 of 183)
ODI/MCID	-	−4.3/−4.2 (−5.1–−3.2)
Δ ODI < −12.8	-	99% (138 of 139)

**Table 5:** Complications according to Clavien–Dindo classification.

Type	Type of Complication	Total Number	First Year *	Later On
I	Nerve root damage without neurological deficite	4	3	1
	Unintended durotomy	1	1	0
Total of type I 6 (3.2%)			5 (2.7%)	1 (0.5%)
II	Hypalgesia L5 contralateral	1	1	0
	L4 neuropathic pain	1	0	1
	Retroperitoneal hematoma (conservative treatment)	1	0	1
	Nerve root damage with paresis in the foot	1	1	0
	Hypesthesia L5	1	0	1
Total of type II 5 (2.7%)			2 (1%)	3 (1.6%)
IIIb	Early disc herniation recurrence (before 90 days after surgery)	4	3	1
	Unresolved nerve root compression (before 90 days after surgery)	2	2	0
	Recurrent disc herniation (90 days after surgery)	4	2	2
	Adjacent segment disease	1	0	1
Total of type III 11 (6%)			7 (3.8%)	4 (2.1%)
Total of complications 22 (12%)			14 (7.6%)	8 (4.3%)

* Within the first year of the introduction of the TESSYS technique in clinical practice.

## Data Availability

Database of our patients is available at Supplementary Materials.

## Supplementary Materials

The following supporting information (Database of our patients) can be downloaded at: https://susy.mdpi.com/user/manuscripts/displayFile/cbe9d704897d2a2428ed01fa5735701a/supplementary.
